# Assessment of robotic telesurgery system among surgeons: a single-center study

**DOI:** 10.1007/s11701-023-01709-5

**Published:** 2023-09-14

**Authors:** Reno Rudiman, Alireza Mirbagheri, Valeska Siulinda Candrawinata

**Affiliations:** 1 Division of Digestive Surgery, Department of Surgery, Faculty of Medicine, Hasan Sadikin Hospital, Universitas Padjadjaran, West Java, Bandung, Indonesia; 2 Department of Medical Physics and Biomedical Engineering, School of Medicine and also Research Center for Biomedical Technologies and Robotics (RCBTR), Advanced Medical Technologies and Equipment Institute (AMTEI), both at Tehran University of Medical Sciences, Tehran, Iran; 3 Department of Surgery, Faculty of Medicine, Pelita Harapan University, Tangerang, Indonesia

**Keywords:** Surgeon performance, Practice duration, Robotic surgery simulator

## Abstract

The field of robotic-assisted surgery is expanding rapidly; therefore, future robotic surgeons will need to be trained in an organized manner. Here, we aimed to examine surgeon performance on the Sinaflex Robotic Telesurgery System for correlation with training hours spent in training program. This is a prospective study of a single-center experience at the Hasan Sadikin Hospital, Bandung City of West Java, Indonesia. We included 43 surgeons from 11 departments, all invited to train using the Sinaflex Robotic Telesurgery system at the Hasan Sadikin Hospital. All study cohorts have never performed a robotic surgery procedure beforehand and have had at least five years of field experience. The surgeons were free to choose their training duration and simulation. After finishing the training session, they were asked to perform several tasks with increasing difficulty levels. There were nine training tasks in total with increasing levels of difficulty. A total of 43 surgeons from 11 different department were included in this prospective study. Our study was separated into 3 different batches and most surgeons failed to pass the examination (*n* = 12, 8, and 9, for batches 1, 2, and 3, respectively). The “failed” surgeon, additionally, tended to be older than the “passed” cohort (49.3 ± 7.4 vs 42.1 ± 7.3 years old, *p* = 0.005). In terms of duration of hours spent training on the robot, there was little difference training hours between the cohort that passed and the cohort that failed cohort (10.0 [8.4–10.1] vs 10.0 [8.0–10.0], respectively) with a *p* value of 0.265. We found no correlation between the total hours spent in the training program and surgeon performance on the Sinaflex robotic telesurgery system. Structured robot surgical training courses must be incorporated into the training programs.

## Introduction

The field of robotic-assisted surgery is expanding rapidly. Most robotic surgery procedures necessitate training, with a typical learning curve eventually leading to reliable performance. Each surgeon who learns the robotic surgical procedures should have their surgical abilities evaluated repeatedly to ascertain where they are on their learning curve. The da Vinci Skills Simulator (dVSS) assesses surgical abilities during robotic surgery [1, 2]. The simulator gives surgeons a variety of tasks in simulated robotic surgery environments and assesses their proficiency using pre-built evaluation criteria.

Virtual reality (VR) simulator advancements portend new training options for robotic surgery. Robotic surgery simulators give beginning robotic surgeons a virtual reality environment to train their surgical techniques without endangering patient safety. Students can practice psychomotor abilities and fundamental procedural skills using current simulators. Additionally, simulator consoles mimic actual robotic surgery consoles to help students become accustomed to the controls [2, 3].

There are currently two hospitals with a robotic surgery training center in Indonesia: Hasan Sadikin Hospital, Bandung and Sardjito Hospital, Yogyakarta. This benefit presents the opportunity of studying intensively about robotic surgery, which is still considered new in Indonesia. One important factor that contributes to surgical outcome is the total years of experience of a surgeon [4]. However, it should be noted that this does not necessarily mean older surgeons possess more experience than their younger counterparts—a notable condition observed in Indonesia. This is an important factor to be analyzed [4, 5]. The primary objective of this study is to assess the association between the duration of robotic surgery training and training success. Training success refers to the surgeon’s proficiency in completing the robotic training modules, determined by their ability to effectively pass the modules. The secondary aim is to determine the obstacles and confounding factors that may affect success in completing the tasks as part of the training.

## Methods

This is a prospective study of a single-center experience at the Hasan Sadikin Hospital, Bandung City of West Java, Indonesia and had gained approval for the study protocol on 26 April 2023. We included 43 surgeons from 11 departments, all invited to train using the Sinaflex robotic telesurgery system (Sina Robotics and Medical Innovators Co., Ltd) at the Hasan Sadikin Hospital. All study cohorts had performed a robotic surgery procedure and were required to have been a practicing surgeon for a minimum of five years of field experience. We gave each participant access to the Sinaflex robotic telesurgery system (Sina Robotics and Medical Innovators Co., Ltd) at the Hasan Sadikin Hospital with a brief tutorial on the controls and exercise principles given by an experienced reference surgeon. The surgeons were free to choose their training duration and simulation. After finishing the training session, they were asked to perform several tasks with increasing difficulty levels. There were nine training tasks in total with increasing levels of difficulty. Each participant was asked to do each job from easiest to most difficult.

1. Camera navigation using the controlling pedals, the operator navigated the camera to match a central target with a colored sphere in 3 dimensions. This task is designed so the operator can obtain the best view as needed in various procedures.
2. 2D path navigation (levels 1, 2, 3): in a two-dimensional view, the operator was required to navigate the camera and follow a target serial.
3. 3D path navigation: In a three-dimensional view, the operator must navigate the camera and follow a target serial.
4. Grasp coordination**:** The operator was instructed to pick up objects with excellent handle and control using the clutch arm.
5. Sorting objects: The operator used the clutch arm to pick up color-coded objects into color-coded containers.
6. Peg placement: More advanced than sorting objects, the operator was asked to pick up a ring from a vertical position into vertical rods.
7. Working with cautery: The operator was tasked to apply dissecting energy to a target, without dissecting nearby structures. This task demands both skills of camera navigation and grasp coordination.
8. Cone placement: The operator used the clutch arm to pick up cones and place them to designated targets, which are more specific than the previous tasks.
9. Object manipulation: The final and most difficult task in this study, in which the operator was tasked to perform an incision along a marked line and complete three stitches through robotic control.

### Data analysis

The statistical analysis was performed using IBM SPSS 26.0 (Statistical Package for the Social Sciences, IBM Corp., Armonk, NY, USA, 2019). The Kolmogorov–Smirnov test was used to determine whether the data had a normal distribution and if the *p* value was higher than 0.05. The median and range suggested that the data were not consistently distributed. We used Mann–Whitney *U* tests to analyze qualitative variables expressed as counts or percentages.

## Results

A total of 43 surgeons from 11 different departments were included in this prospective study. Our study was separated into three batches, and most surgeons failed to pass the module (*n* = 12, 8, and 9, for batches 1, 2, and 3, respectively). The “failed” surgeon, additionally, tended to be older than the “passed” cohort (49.3 ± 7.4 vs 42.1 ± 7.3 years old, *p* = 0.005). Most surgeons who failed were from the Department of Obstetrics and Gynaecology (*n* = 6; 21%). All surgeons from the neurosurgery, orthopedics, and vascular surgery failed, whereas all plastic surgeons and general surgeons passed the test. There was little difference in training hour duration between passed and failed cohorts [10.0 [8.4–10.1] vs 10.0 [8.0–10.0], respectively), and time on the trainer was deemed not statistically significant with a *p* value of 0.265. Results are summarized at Table 1.

**Table 1:** Demographic characteristics of included surgeons from various department (*n* = 43)

Variable	Status		*p* value
	Failed	Passed	
Age, years old	49.3 ± 7.4	42.1 ± 7.3	0.005
Batch			0.200
1	12 (66.7)	6 (33.3)	
2	8 (53.3)	7 (46.7)	
3	9 (90.0)	1 (10.0)	
Departments			0.186
Ob/Gyn	6 (75.0)	2 (25.0)	
CTVS	1 (25.0)	3 (75.0)	
Digestive	4 (100.0)	0 (0.0)	
Pediatric	3 (75.0)	1 (25.0)	
Plastic	0 (0.0)	1 (100.0)	
NS	4 (100.0)	0 (0.0)	
ENT	5 (62.5)	3 (37.5)	
Urology	3 (50.0)	3 (50.0)	
General surgery	0 (0.0)	1 (100.0)	
Orthopedic	2 (100.0)	0 (0.0)	
Vascular	1 (100.0)	0 (0.0)	
Training duration, hours	10.0 (8.0–10.0)	10.0 (8.4–10.1)	0.265

*Ob/Gyn* obstetrics and gynecology, *CTVS* cardiothoracic vascular, *NS* neurological surgery, *ENT* ear, nose, and throat

## Discussion

We found no statistical significance between the duration of training hours of the failed and passed cohorts from various surgical departments. However, the age of a surgeon was found to be significant statistically (*p* = 0.005). Between departments, it was observed that there was a higher passing rate for the department of vascular surgery, plastic surgery, and general surgery. An equal passing rate was observed in the urology department. From three batches, most study participants failed to pass the examination. This is the first study to examine the correlation between training hours and surgeon performance.

### Rationale of the study

Programs for robotic surgery offer a customizable approach to education for fundamental robotic-assisted surgical training [1, 6]. A significant demand on one’s schedule is frequently caused by the nature of trainees who attempt to integrate patient care with educational opportunities. As a result, allocating additional training time for each learner within a study program may be challenging. Telesurgery programs’ accessibility allows scholars to self-plan their education and better develop the information and abilities necessary for robotic-assisted procedures.

Despite the da Vinci Technology Training Pathway offering videos and proficiency checklists to guide students in refining their psychomotor and procedural surgical robot skills, most trainees failed in the current study [1–3, 6]. These programs, however, need more professional mentoring, and there needs to be more real-time performance feedback [7]. Lack of real-time performance feedback may cause trainees to develop ineffective motor patterns, hindering their learning of surgical skills [8, 9].

In addition, familiarity with the teaching platform and a lack of psychomotor abilities are two initial challenges that new robotic surgeons must overcome; however, these can be solved through simulated training [10]. Particularly in the early stages of skill acquisition, understanding the fundamental operations of the robotic console enables the surgeon to concentrate more on the patient and the surgical process being carried out. Additionally, surgical simulation improves the effectiveness of the surgeon. Before patient interaction, simulation training can improve psychomotor skills and platform familiarity, according to an initial construct validity investigation of the dVSS [11–13].

### Real life applicability

The majority of robotic curricula emphasize robotic surgery's technical components. However, surgery needs more than just using one's psychomotor abilities. Independent practice requires procedural knowledge, which includes an understanding of the anatomy, the natural history of the disease, indications for a procedure, the steps, and probable consequences [2]. Robotic programs should cover the procedural knowledge domain, or they should be covered as part of a more extensive surgical training curriculum [14, 15]. It has recently been developed for publicly recorded videos to evaluate technical skills objectively and to provide benchmarks and criticism. With these cutting-edge tools, students may evaluate their performance via an online portfolio, comprehend their learning schedule, and compare their abilities to those of the best students in the class [15].

Solid knowledge, effective communication abilities, clinical judgment, and technical proficiency are necessary for high-quality surgical outcomes. We found that older trainees tend to fail in the exam than their junior. This can be argued due to the low technology literacy and slower adaption of older surgeon in Indonesia [16]. However, considering the unremarkable difference between cohorts (49.3 ± 7.4 vs 42.1 ± 7.3 years old). This is only 7-year difference and might have little or no difference in the digital literacy or adaptation.

### Future suggestions

In the future, a step-by-step progression through a curriculum, specifically through procedural procedures while at the console, will be among the best practices. A crucial component of learning is feedback. Compared to unstructured training programs, proctoring during organized training programs increases skill gains; hence it should be included in the curriculum. Future standards are anticipated to take proficiency gained through prolonged training into account. Tools like the Robotics Objective Structured Assessment of Technical Skills, which has been validated, could provide a benchmark between simulations and live console practice [17, 18]. Additionally, the Global Evaluative Assessment of Robotic Skills, verified, may be used in an operational scenario [19].

### Limitations

Our limitation is that we did not use any equivalent rubric to assess surgical performance from every department. Second, we did not examine operating timelines and complication rates in procedures for a single trainee. Another drawback is that the robotic curriculum must assess non-technical abilities like leadership, risk management, teamwork, communication, and situational awareness. It is becoming more apparent how frequently unfavorable outcomes are brought on by actions taken away from the operating console.

## Conclusions

We found no correlation between the total hours spent in the training program and surgeon performance on the Sinaflex robotic telesurgery system. The increasing uptake of the robotic surgical platform suggests that it will continue to be the surgical method of choice for many challenging surgical surgeries. Structured robot surgical training courses must be incorporated into the training programs. The precise measurements and results required for review and credentialing are still in development. However, integrating robotics into a training program requires an organized approach to online learning, practice on simulators, progressive intra-operative experience, and controlled case-by-case evaluation.

## Data Availability

Not applicable.
